# Voluntary medical male circumcision (VMMC) for prevention of heterosexual transmission of HIV and risk compensation in adult males in Soweto: Findings from a programmatic setting

**DOI:** 10.1371/journal.pone.0213571

**Published:** 2019-03-07

**Authors:** Hillary Mukudu, Janan Dietrich, Kennedy Otwombe, Mmatsie Manentsa, Khuthadzo Hlongwane, Maetal Haas-Kogan, Benn Sartorius, Neil Martinson

**Affiliations:** 1 Perinatal HIV Research Unit (PHRU), Faculty of Health Sciences, University of the Witwatersrand, Johannesburg, South Africa; 2 School of Public Health, Faculty of Health Sciences, University of Witwatersrand, Johannesburg, South Africa; 3 Harvard Global Health Institute and Harvard University Center for AIDS Research, Harvard University, Cambridge, United States of America; 4 Department of Public Health Medicine, School of Nursing and Public Health, University of KwaZulu-Natal, Durban, South Africa; University of Oxford, UNITED KINGDOM

## Abstract

**Background:**

Clinical trials have clearly shown a reduction in HIV acquisition through voluntary medical male circumcision (VMMC). However, data assessing risk compensation under programmatic conditions is limited.

**Methods:**

This was a prospective cohort of HIV seronegative males aged 18–40 years receiving VMMC between November 2012 and July 2014. HIV serostatus was determined pre and post VMMC. Risk compensation was defined as a decrease in condom use at last sex act and/or an increase in concurrent sexual relationships, both measured twelve months post-circumcision.

**Results:**

A total of 233 males were enrolled and underwent voluntary medical male circumcision (VMMC) for prevention against HIV. There was no evidence of risk compensation post-circumcision as defined in this study. Significant increases in proportion of participants in the 18–24 years age group who knew the HIV status of their sexual partner (39% to 56%, p = 0.0019), self-reported condom use at last sex act (21% to 34%, p = 0.0106) and those reporting vaginal sexual intercourse in the past 12 months (67% to 79%, p-value = <0.0001) were found. In both 18–24 and 25–40 years age groups, there was a significant increase in perception of being at risk of contracting HIV (70% to 84%, p-value = <0.0001).

**Conclusion:**

No significant risk compensation was observed in this study on comparing pre-and post-circumcision behaviour. An increase in proportion of participants in the 18–24 years age group who had vaginal intercourse in the first 12 months post-circumcision as a possibility of risk compensation was minimal and negated by an increase in proportion of those reporting using a condom at the last sex act, increase in knowledge of partner’s HIV status and lack of increase in alcohol post-circumcision.

## Introduction

Scale up of Voluntary Medical Male Circumcision (VMMC) in South Africa followed recommendations by the World Health Organization (WHO) for the roll-out of VMMC in 2007 as an added HIV prevention strategy in countries with a generalized HIV epidemic. By 2016, nearly 15 million VMMCs had been performed in 14 priority countries of Sub-Saharan Africa [1]. VMMC has been shown to decrease HIV infection under research conditions[2] [3]. However, data on risk compensation after circumcision in a programme setting is limited. Furthermore, evidence of risk compensation in males aged 24–40 years, the group with the highest HIV incidence in South Africa, also remains unknown [3].

There are concerns over the potential increase in high risk sexual behaviour as a result of risk compensation particularly in high HIV incidence age groups [4] [5][6] which may mitigate the effectiveness of circumcision in preventing new HIV infections. Risk compensation is defined as modification of behaviour as a result of changes in the perceived risk of HIV infection[6]. With respect to HIV and circumcision, this would manifest itself in circumcised males knowing that they are less likely to contract HIV than before circumcision and in response, they might increase high-risk sexual behavior. In a 2013 study done in Uganda on male circumcision that assessed sexual behavior and HIV status, it was found that circumcised men are more likely to engage in risky behavior than their uncircumcised counterparts[7]. In contrast, another study done in Kenya found no evidence of risk compensation among males at least 24 months post-circumcision compared to the uncircumcised[8]. Also, a recent comparison of three cross-sectional studies showed an absence or minimal risk compensation [9].

This study sought to further explore and elucidate the effect of VMMC on risky behavior post-circumcision in a programme setting [10] [11].

## Methods

The study was conducted at a high volume VMMC clinic based at Chris Hani-Baragwanath Academic Hospital in Soweto, a peri-urban township in South Africa. A prospective cohort of men requesting VMMC was established, inviting every third HIV-seronegative male, aged 18–40 years who reported living in Soweto from November 2012 to July 2014 to participate. Men were circumcised routinely and followed up prospectively thereafter. A post-circumcision visit was scheduled 12 months after VMMC.

An original target sample size of 904 was set to satisfy the primary objective of the study which was to determine a 50% difference in one-year HIV sero-incidence between before and after circumcision at a power of 80%, assuming a 15% non-informative loss to follow-up after VMMC in adult males. However, due to funding constraints and logistical challenges, HIV incidence was not reported on, and of the 496 participants enrolled, only follow-up data from 233 of these was utilised for the risk compensation analysis reported here.

Selected VMMC-trained staff of the clinic were trained and conducted all study procedures. A structured questionnaire focused on participants’ recall of HIV-risk behavior in the 12 months period pre-VMMC was conducted at enrolment. Surgical MMC was then performed on participants according to WHO guidelines [12]. During the study follow-up period, participants were contacted monthly by telephone, to remind them of post-circumcision study visits. At a final study exit visit conducted 12 months post-VMMC, participants were tested for HIV using a rapid test and the structured questionnaire on risky behaviours was re-administered. Double data entry, was used to capture data into a REDCap database [13]. Questionnaires used were standardised and pilot tested prior to study commencement in October 2012.

The primary outcome of interest was HIV serostatus at the post-VMMC visit. Due to the rare occurrence of HIV incident infection over the study time frame ~ (3 sero-conversions in this case), HIV risk was represented by condom use at last sex act and concurrent sexual partnerships. These two variables were secondary outcomes of the study. Condom use at last sex act was defined as using a condom during the most recent sex act and concurrent sexual partnerships was defined as having more than one sexual partner at the time of the interview. We also measured perception of HIV risk as a possible confounder of concurrent sexual partnerships and condom use at last sex act. We defined perception of HIV risk as participant feeling that they were personally at risk of contracting HIV. Other confounders measured were having vaginal sexual intercourse in the last 12 months, a history of treatment of sexually transmitted infections (STIs) in the last 12 months, knowledge of sexual partners’ HIV status at the last sex act and having taken alcohol at the time of last sexual act.

## Statistical analysis

Risk compensation was defined as a decrease in condom use at last sex act and or increase in concurrent sexual partners after circumcision. Selection of these two factors is based on the fact that concurrent sexual partnerships are main drivers behind high prevalence of HIV in sub-Saharan Africa among males. Furthermore, condom use is an effective mode of HIV prevention[14][15][16]. The association between categorical measures before and after circumcision was assessed using the McNemar’s test.

## Ethical considerations

Study protocol was approved by the University of the Witwatersrand Human Research Ethics Committee (certificate #M120634). Chris Hani-Baragwanath Hospital Research Committee provided additional approval for the study. Strict confidentiality procedures were maintained and written informed consent was obtained from all participants. The study protocol can be accessed at: http://dx.doi.org/10.17504/protocols.io.seiebce.

## Results

### Demographics

**Fig 1:**
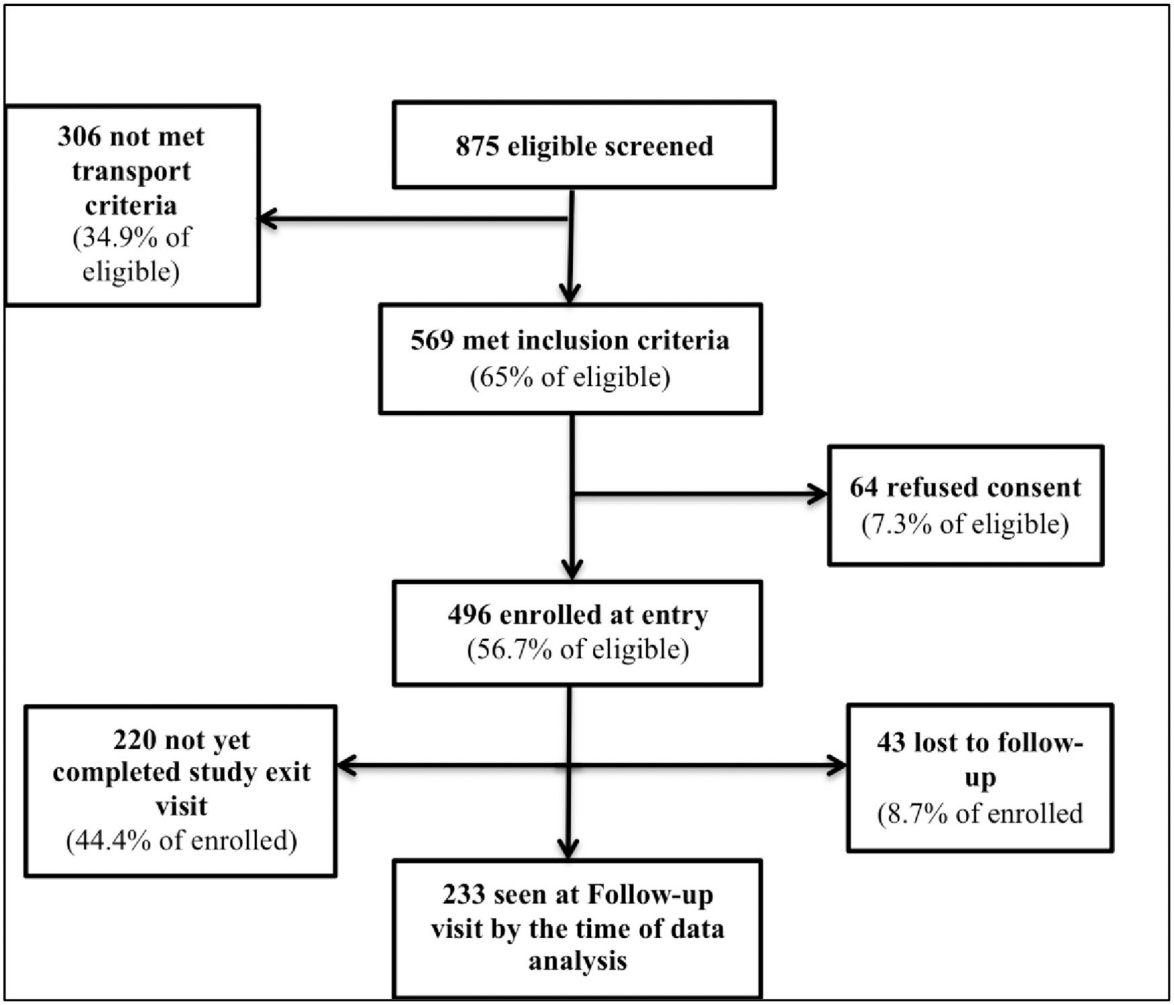
Screening, enrolment and follow-up of study participants.

The study enrolled 496 men, of whom 233 completed the 12-month follow-up visit (Fig 1 Study-flow).

Distribution of baseline characteristics is shown in Table 1. At sexual debut, higher condom use was reported for 18–24 years age group compared to 25–40 years age group (58% vs. 45%, p-value = 0.0469). More participants in 25–40 years age group had sexual intercourse in the previous twelve months compared to those in the 18–40 years age group (90% vs. 67%, p-value <0.0001). A higher proportion of 25–40 years olds compared to 18–24 years olds also reported knowledge of partner’s HIV status (71% vs. 39%, p-value = <0.0001) and use of condoms at the last sexual act (57% vs. 21%, p-value = <0.0001).

**Table 1:** Baseline demographic characteristics by age-group.

Variables	Overall	18–24 years	25–40 years	P-Value
**Number followed at 12 months**	233	113	120	
**Marital status**				
Married/living together with partner n(%)	33 (14)	5 (4)	28 (23)	**< .0001**
Single/ Not married or living together n(%)	200 (86)	108 (96)	92 (77)	
**Occupational status**				
Employed n(%)	104 (45)	29 (26)	75 (63)	**< .0001**
Student n(%)	57 (24)	53 (47)	4 (3)	
Unemployed n(%)	72 (31)	31 (27)	41 (34)	
**Education**				
High/Primary school n(%)	167 (72)	75 (66)	92 (77)	0.0813
Tertiary n(%)	66 (28)	38 (34)	28 (23)	
**Reason for VMMC**				
Prevention against HIV/STIs n(%)	158 (68)	81 (72)	77 (64)	0.2198
Other n(%)	75 (32)	32 (28)	43 (36)	
**Had anal sex within past six months**				
No n(%)	224 (96)	108 (96)	116 (97)	0.7427
Yes n(%)	9 (4)	5 (4)	4 (3)	
**Ever had vaginal sex**				
No n(%)	12 (5)	12 (11)	0 (0)	-
Yes n(%)	221 (95)	101 (89)	120 (100)	
Age for debut sex*				
<16 years n(%)	59 (27)	29 (29)	30 (25)	0.5342
≥16 years n(%)	162 (73)	72 (71)	90 (75)	
Condom use for debut sex*				
No n(%)	108 (49)	42 (42)	66 (55)	**0.0469**
Yes n(%)	113 (51)	59 (58)	54 (45)	
**Had vaginal sex within past 12 months**				
No n(%)	49 (21)	37 (33)	12 (10)	**< .0001**
Yes n(%)	184 (79)	76 (67)	108 (90)	
**Had alcohol before sex with main partner**				
No n(%)	196 (84)	99 (87)	97 (80)	0.157
Yes n(%)	37 (16)	14 (12)	23 (19)	
**Knowledge of partner’s HIV status**				
No n(%)	104(35)	69 (61)	35 (29)	**< .0001**
Yes n(%)	129 (55)	44 (39)	85 (71)	
**Concurrent sexual partners**				
No n(%)	163 (70)	77 (68)	86 (72)	0.9104
Yes n(%)	70 (30)	36 (32)	34 (28)	
**Condom use at last sex act**				
No n(%)	141 (61)	89 (79)	52 (43)	**< .0001**
Yes n(%)	92 (39)	24 (21)	68 (57)	
Perception of HIV risk				
No n(%)	71 (30)	41 (36)	30 (25)	0.149
Yes n(%)	162 (70)	72 (64)	90 (75)	

*Totals may not be equal to the sample size as a result of missing values

Participants lost to follow-up or not yet completed 12 months visit did not differ from those retained in the study in relation to race, age, age at sexual debut, history of anal intercourse, condom use at last sex act, transactional sex, use of alcohol with sexual intercourse, risky sexual relationships, knowledge of sexual partners HIV status, and concurrent sexual partnerships (all p-values >0.05). However, those who completed follow-up were less likely to have used intravenous drugs (0% vs. 3%, p = 0.005) and less likely to have a history of an STI (2% vs 14%, p<0.001) (Table 2).

**Table 2:** Comparison of participants retained in the study and lost to follow-up.

Risk Factor	Followed- (n = 233)	Lost or not yet completed (n = 263)	p-value
Race: African n(%)	233 (100)	260 (99)	0.280*
Age (mean years) (SD)	25.5 (5.4)	25.6 (5.3)	0.5823 ^†^
Age at Sexual Debut (mean years) (SD)	16.6 (2.3)	16.8 (2.6)	0.8163 ^†^
Intravenous drug use n(%)	0 (0)	8 (3)	0.005*
History of STIs treatment in past 12 months n(%)	4 (2)	37 (14)	<0.0001*
Concurrent sexual partners n(%)	70 (30)	74 (28)	0.641 ^‡^
Condom Use at last sex act n(%)	92(39)	97 (37)	0.587 ^‡^
Transactional Sexual Intercourse n(%)	3 (1)	0 (0)	0.103 *
Use of alcohol with sexual intercourse n(%)	37 (16)	50 (19)	0.360 ^‡^
Knowledge of Partner’s status n(%)	129 (55)	139 (53)	0.575 ^‡^
Perception of HIV risk n(%)	162 (70)	181(69)	0.865^‡^

* Fisher’s test

^†^t-test

^‡^ Chi^2^ test

#### Risky behaviours pre and post-circumcision

The distribution of risky behaviours pre- and post VMMC is shown in Table 3. On comparing pre- to post-circumcision, there was a significant increase in the proportion of males engaging in sexual intercourse in the preceding twelve months from 184 (79%) to 204 (88%) (p-value = 0.0130), knowing the HIV status of sexual partner from 129 (55%) to 152 (65%) (p-value = 0.0063) and a perception of being at risk of contracting HIV from 162 (70%) to 195 (84%) (p-value = <0.0001). There was also a significant increase in males using a condom at last sex act from 92(40%) to 112 (48%) (p-value = 0.0168).

**Table 3:** Pre and post-circumcision by sexual behaviours.

Variables	Before circumcision	After circumcision	P-Value
**Number followed at 12 months**	233	233	
**Had vaginal sex within past 12 months**			
No (%)	49 (21)	29 (12)	**0.0130**
Yes (%)	184 (79)	204 (88)	
**Had alcohol before sex with at previous sexual intercourse**			
No (%)	196 (84)	203(87)	0.3550
Yes (%)	37 (16)	30 (13)	
**Knowledge of partner’s HIV status**			
No (%)	104 (45)	81 (35)	**0.0063**
Yes (%)	129 (55)	152 (65)	
**Concurrent sexual partners**			
No(%)	163 (70)	162 (70)	0.0910
Yes (%)	70(30)	71 (30)	
**Condom use at last sex act**			
No(%)	141 (60)	121(52)	**0.0168**
Yes(%)	92 (40)	112 (48)	
**Perception of HIV risk**			
No(%)	71 (30)	38 (16)	**< .0001**
Yes (%)	162 (70)	195 (84)	

#### Comparison of HIV risk behaviour before and after circumcision between age groups

A comparison of pre- and post-circumcision risky sexual behaviours stratified by age group (18–24 vs. 25–40 years) is shown in Table 4. There was an increase in proportion of men engaging in sexual intercourse for the 18–24 years age group. Similarly, knowledge of partners HIV status and condom use at last sex act was increased in the 18–24 age groups. In both age groups there was a significant increase in perception of being at risk of contracting HIV.

**Table 4:** Sexual behaviours by age group—Before and 12 months after circumcision.

Variables	Before circumcision	After circumcision	Odds Ratio 95% Confidence Interval	P-Value
**Number followed at 12 months**	233	233		
**Concurrent sexual partners**				
18–24 Years n(%)	36 (32)	40 (35)	1.2 (0.6–2.4)	0.6358
25–40 Years n(%)	34 (28)	31 (26)	0.9 (0.4–1.7)	0.7493
Overall n(%)	70 (30)	71 (30)	1.0 (0.6–1.6)	0.9104
**Had vaginal sex within past 12 months**				
18–24 Years n(%)	76 (67)	89 (79)	**2.3 (2.0–5.2)**	**< .0001**
25–40 Years n(%)	108 (90)	115 (96)	21.6 (9.0–67.9)	< .0001
Overall n(%)	184 (79)	204 (88)	**2.7 (1.3–5.7)**	**0.0037**
Had alcohol before sex at last sexual intercourse*				
18–24 Years n(%)	14 (12)	17(15)	1.3 (0.5–3.6)	0.6636
25–40 Years n(%)	23 (19)	13 (11)	0.4 (0.2–1.1)	0.0755
Overall n(%)	37 (16)	30 (13)	0.7 (0.4–1.4)	0.3817
Knowledge of partner’s HIV status*				
18–24 Years n(%)	44 (39)	63 (56)	**3.4 (1.5–8.6)**	**0.0019**
25–40 Years n(%)	85 (70)	89 (74)	1.25 (0.6–2.6)	0.6177
Overall n(%)	129 (55)	152 (65)	**2.0 (1.2–3.3)**	**0.0086**
**Condom use at last sex act**				
18–24 Years n(%)	24 (21)	38 (34)	**2.8 (1.2–7.1)**	**0.0106**
25–40 Years n(%)	52 (43)	74 (62)	1.4(0.7–2.7)	0.4296
Overall n(%)	**92 (39)**	**112 (48)**	**0.6 (0.3–0.9)**	**0.0225**
**Perception of HIV risk**				
18–24 Years n(%)	72 (64)	91 (81)	**3.4 (1.5–8.6)**	**0.0019**
25–40 Years n(%)	90 (75)	104 (87)	**3.8 (1.4–13.0)**	**0.0066**
Overall n(%)	162 (70)	195 (84)	**3.5 (1.9–7.1)**	**<0.0001**

*Totals may not be equal to the overall as a result of missing values

## Discussion

Our study’s findings corroborate evidence of absence of risk compensation after VMMC [17][11]. Our study also illuminated possible additional benefits of VMMC programs specifically the HIV counselling and VMMC education sessions. It was found that post-circumcision participants in the 18–24 years age group were more likely to know the HIV status of their sexual partner and more likely to use a condom at the last sex act [18]. Absence of similar findings in the 25–40 years age will warrant further research considering that older men are at substantially higher HIV risk [3].

Our study also found that participants in the 18–24 years age group were more likely to engage in sexual intercourse after VMMC than before. This is in line with findings in other parts of Africa [11]. It could be explained by the fact that VMMC has been linked to sexuality and masculinity in some parts of South Africa which has increased uptake of the program [19] but also that the cohort had aged by a year.

These findings show that there is little or no change in risky sexual behaviour after circumcision, particularly absence of evidence for an increase in concurrent sexual partnerships is re-assuring, specifically in the context of prior research suggesting that concurrent sexual partnerships are a main driver behind the high prevalence of HIV among males in sub-Saharan Africa. [16].

The increase in the proportions having sexual intercourse post-circumcision are not large and are not by themselves risky as evidenced by increase in condom use, knowledge of partner’s HIV status and lack of increase in alcohol use.

Limitations of this study include the high dropout rate as evidenced by less than half of participants completing the 12-month study visit.

Data on risk factors was self-reported allowing for social desirability bias for which participants may have responded in a favourable manner. Having a single data point 12 months post-circumcision to collect data, may have introduced recall bias. Selection bias was introduced to the study by the inclusion criteria and having those who completed the 12 months follow-up visit being more likely to have used intravenous drugs and less likely to have a history of an STI. This limited generalizability of study findings as a representation of the general population with respect to age group, HIV status and those not seeking VMMC. Also, a lack of a control group which should have been uncircumcised males limited the generalizability of certain findings.

Effectiveness of VMMC must take into account changes in risk behaviours as well as possible effects of risk compensation. Further exploration must be undertaken to investigate why there was a significant increase or decrease in specific risk, and protective, behaviours post VMMC, and the relationship between risk behaviours and risk compensation.

## Conclusion

In this cohort we found in the 12 months post-circumcision compared to pre, participants in the 18–24 years age groups, were more likely to use a condom use at last sex act, more likely to know the HV status of sexual partner and more men (both 18-24- and 25-40-years age groups) considered themselves at risk of HIV infection. This suggests that there are other potential benefits conferred by VMMC in a programme setting as a biomedical and behavioural intervention for the prevention of HIV transmission.
